# Molecular metal–N_*x*_ centres in porous carbon for electrocatalytic hydrogen evolution

**DOI:** 10.1038/ncomms8992

**Published:** 2015-08-07

**Authors:** Hai-Wei Liang, Sebastian Brüller, Renhao Dong, Jian Zhang, Xinliang Feng, Klaus Müllen

**Affiliations:** 1 Max-Planck-Institute for Polymer Research, Ackermannweg 10, 55128 Mainz, Germany; 2 Department of Chemistry and Food Chemistry & Center for Advancing Electronics Dresden (cfaed), Technische Universität Dresden, Mommsenstrasse 4, 01062 Dresden, Germany

## Abstract

Replacement of precious platinum with efficient and low-cost catalysts for electrocatalytic hydrogen evolution at low overpotentials holds tremendous promise for clean energy devices. Here we report a novel type of robust cobalt–nitrogen/carbon catalyst for the hydrogen evolution reaction (HER) that is prepared by the pyrolysis of cobalt–N_4_ macrocycles or cobalt/*o*-phenylenediamine composites and using silica colloids as a hard template. We identify the well-dispersed molecular CoN_*x*_ sites on the carbon support as the active sites responsible for the HER. The CoN_*x*_/C catalyst exhibits extremely high turnover frequencies per cobalt site in acids, for example, 0.39 and 6.5 s^−1^ at an overpotential of 100 and 200 mV, respectively, which are higher than those reported for other scalable non-precious metal HER catalysts. Our results suggest the great promise of developing new families of non-precious metal HER catalysts based on the controlled conversion of homogeneous metal complexes into solid-state carbon catalysts via economically scalable protocols.

## Abstract (web summary)

Hydrogen evolution from water promises a future clean energy source, however the cost of noble metal catalysts, which are necessary for high efficiency, are very expensive. Here, the authors fabricate a porous cobalt–nitrogen/carbon catalyst which can deliver high activity and stability but at reduced cost.

## Graphical abstract

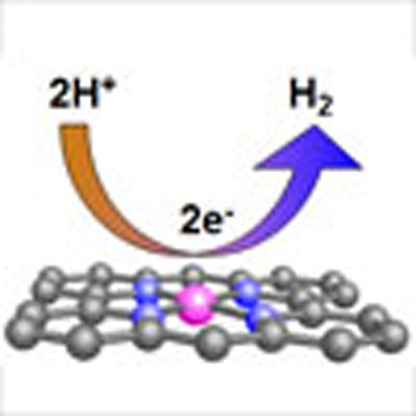

To meet the scientific challenges of the increasing global demand for energy and diminishing sources of fossil fuels, great efforts have been devoted to exploit alternative and renewable energy sources and carriers. The clean and efficient production of hydrogen through electrocatalytic processes, such as photoelectrochemical water splitting or electrolysis coupled to renewable energy sources, represents a promising and appealing solution as a next-generation technology for sustainable energy conversion^1^,^2^,^3^. The development of active hydrogen evolution reaction (HER) electrocatalysts with low overpotentials is crucial for the successful implementation of water splitting technologies. Although platinum-based catalysts have proven as the most efficient electrocatalysts for HER, the prohibitive cost and scarcity of platinum significantly hinder its widespread technological use. It is thus highly desirable to develop low-cost catalyst systems based on earth-abundant elements that are capable of producing hydrogen from water with high catalytic activity and stability^4^,^5^,^6^,^7^,^8^,^9^,^10^.

Inspired by the naturally occurring hydrogen evolution and uptake on hydrogenase enzymes, chemists have made great breakthroughs in recent years on the development of bioinspired, synthetic metal complex HER catalysts, including cobaloxime^7^,^11^, cobalt diimine–dioxime^12^ and nickel phosphane compounds^13^, which are molecularly similar to the Fe–Fe and Fe–Ni active sites of hydrogenases. Unfortunately, most bioinspired catalysts suffer from instability in aqueous solution and require large overpotentials to achieve appreciable turnover frequencies (TOFs).^12^,^13^,^14^,^15^ The practical utilization of these synthetic molecular catalysts in aqueous electrolytes therefore requires their careful grafting onto carbon nanotubes by additional and complex chemical processes^14^,^15^. In contrast, independent efforts in solid-state electrocatalysis have led to several efficient inorganic crystalline catalysts, such as transition metal dichalcogenides^16^,^17^,^18^,^19^,^20^,^21^,^22^,^23^,^24^, phosphides^25^,^26^,^27^, carbides^28^,^29^,^30^,^31^, nitrides^32^,^33^, borides^28^ and metallic nanoparticles embedded in carbon.^34^,^35^ Despite their promising performance, it is highly challenging to control the atomic-scale surface structure of these inorganic materials and to preferentially expose a greater fraction of their active sites, for example, the edge sites of transition metal dichalcogenides^21^,^23^.

In this study, we demonstrate an up-scalable, solid-state, carbon-based catalyst for HER with molecular CoN_*x*_ active sites that are structurally similar to molecular metal–N_4_ macrocycle catalysts. The CoN_*x*_/C catalysts are prepared by a straightforward pyrolysis process at high temperature in which cobalt–N_4_ macrocycles or cobalt/*o*-phenylenediamine composites are used as precursors and silica colloid is used as template. The achieved catalysts exhibit a high specific surface area (1,074 m^2^ g^−1^) and well-dispersed, highly active CoN_*x*_ active sites. In strong acid electrolytes (for example, 0.5 M H_2_SO_4_), the CoN_*x*_/C catalyst shows an overpotential of only 133 mV at a current density of 10 mA cm^−2^. Moreover, acid leaching and thiocyanate ion poisoning experiments identify that the well-dispersed molecular CoN_*x*_ centres in carbon are responsible for the outstanding HER performance of the CoN_*x*_/C catalysts. Importantly, the CoN_*x*_/C catalysts exhibit unprecedented TOFs (TOFs per Co atom of 0.39 and 6.5 s^−1^ at an overpotential of 100 and 200 mV, respectively), which are superior to those of the recently reported breakthrough HER catalysts based on biomimetic molecules^14^,^15^, metal dichalcogenides^18^,^21^ and phosphides^25^,^26^,^27^ (TOF<1.0 s^−1^ at 200 mV overpotential).

## Results

### Electrocatalyst preparation

**Figure 1:**
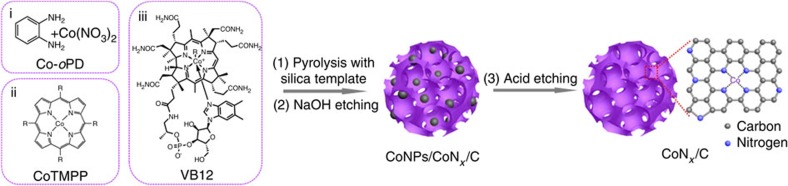
Schematic illustration of the synthesis of the CoN_*x*_/C electrocatalysts. (1) Mixing of carbon, nitrogen, and cobalt precursors (Co-*o*PD, CoTMPP, or VB12) with the silica template and pyrolyzing of the mixture; (2) NaOH etching to remove the silica template and form CoNPs/CoN_*x*_/C composites; (3) Acid etching with H_2_SO_4_ to remove Co-containing nanoparticles and formation of carbon-based catalysts containing CoN_*x*_ sites. The second and third heat treatments after the NaOH and H_2_SO_4_ etching steps are not shown.

A schematic diagram describing the catalyst preparation is shown in Fig. 1. Three different precursors were examined in this work for the construction of the CoN_*x*_/C catalysts, including metal–N_4_ macrocycles, that is, cobalt tetramethoxyphenylporphyrin (CoTMPP) and vitamin V12 (VB12), as well as the cobalt/*o*-phenylenediamine composites (Co-*o*PD). To improve the porosity of the carbon-based catalysts, silica nanoparticles were used as hard template during the synthesis^36^,^37^. Typically, a mixture of C/N/Co precursors and silica colloids (HS40, 12 nm) was first subjected to pyrolysis at different temperatures in the range of 600–1,000 °C under N_2_ atmosphere. The pyrolyzed product was then etched in a 2.0 M NaOH solution to remove the silica template. The NaOH-treated sample was next treated in 0.5 M H_2_SO_4_ at 90 °C for 4 h to leach out unstable, metallic cobalt nanoparticles. Two additional heat treatments at the same temperature as for the pyrolysis step were carried out after the NaOH and H_2_SO_4_ etching. During the pyrolysis of the metal complex, the precursors decomposed and formed carbon-based materials integrated with active CoN_*x*_ moieties. The pyrolysis temperature was optimized for each precursor according to its HER activity (Supplementary Fig. 1). We found that the Co-*o*PD catalyst pyrolyzed at 900 °C yielded the highest HER activity (Supplementary Fig. 2). Therefore, the CoN_*x*_/C catalysts discussed below were all produced from Co-*o*PD at 900 °C unless otherwise specified. For comparison, metal-free N/C, nitrogen-free Co/C and pure carbon catalysts were also prepared (details of the fabrication process for all materials are given in the Methods section).

### Electrocatalytic performance for HER

**Figure 2:**
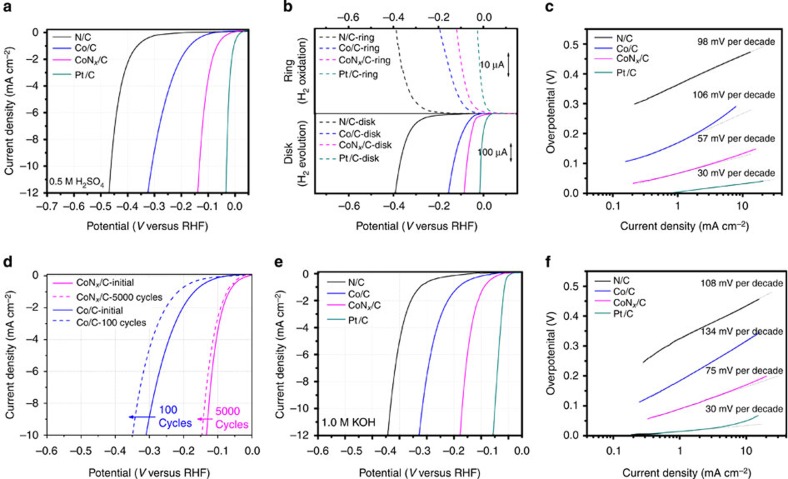
Electrocatalytic performance of the CoN_*x*_/C electrocatalysts. (**a**) HER polarization plots of the CoN_*x*_/C, N/C, Co/N and Pt/C catalysts in 0.5 M H_2_SO_4_. (**b**) RRDE measurements of hydrogen evolution from a 0.5 M H_2_SO_4_ solution on different catalyst-modified electrodes. The Pt-ring electrode was maintained at 0.7 V for the oxidation of the H_2_ that was evolved on the disk electrode. (**c**) Tafel plots obtained from the polarization curves in **a**. (**d**) Initial and post-potential cyclic voltammograms of CoN_*x*_/C (5000 cycles) and Co/N (100 cycles) in 0.5 M H_2_SO_4_. Potential sweeps were cycled between 0.2 and −0.25 V versus RHE (not iR-corrected). (**e**) HER polarization plots of CoN_*x*_/C, N/C, Co/N and Pt/C catalysts in 1.0 M KOH. (**f**) Tafel plots obtained from the polarization curves in **e**. For all RDE and RRDE measurements, the catalyst loading is 2.0 mg cm^−2^ for non-Pt materials and 0.2 mg cm^−2^ (40 μg_Pt_ cm^−2^) for the Pt/C catalyst. For CoN_*x*_/C catalysts, the cobalt loading is only 2.8 μg_Co_ cm^−2^, based on the ICP-AES measurements. Electrode rotation speed: 1,600 r.p.m.; scan rate: 5 mVs^−1^.

The HER performance of the CoN_*x*_/C catalysts and reference samples, including N/C, Co/C and a commercially available Pt/C (20 wt% Pt, BASF), was first evaluated using the rotating disk electrode (RDE) technique in an Ar-saturated 0.5 M H_2_SO_4_ solution. As shown in Fig. 2a, metal-free nitrogen-doped carbon (N/C) can act as a HER catalyst, although the overpotential is quite high (460 mV at 10 mA cm^−2^). According to previous reports, nitrogen doping could reduce the Gibbs free energy of hydrogen adsorption and result in an improved HER activity compared with pure carbon materials^38^. The Co/C catalyst exhibited higher HER activity than the metal-free N/C catalyst and the pure carbon material (Supplementary Fig. 3); it had an overpotential of 310 mV at a current density of 10 mA cm^−2^, which is comparable to those of the Fe/Co nanoparticle-based catalysts that were recently reported^34^,^35^. The simultaneous incorporation of nitrogen and cobalt into carbon led to a profound enhancement of the HER activity, as reflected by the large shift of the polarization curve of the CoN_*x*_/C catalyst to a lower overpotential compared with the N/C and Co/C catalysts (Fig. 2a). In particular, the CoN_*x*_/C catalyst showed an overpotential of only 133 and 156 mV at a current density of 10 and 20 mA cm^−2^ (after ohmic and capacitive corrections, see Supplementary Fig. 4 for details), respectively, which are only ∼100 mV lower than the benchmark Pt/C catalyst (overpotential of 32 mV at 10 mA cm^−2^). Moreover, the overpotential of the CoN_*x*_/C catalyst is much lower than the values reported for most acid-stable, earth-abundant, molecular and inorganic HER electrocatalysts (Supplementary Table 1), including CNTs-supported nickel bisdiphosphine complexes (300 mV overpotential at 4 mA cm^−2^)^14^ and cobalt diimine–dioxime complexes (590 mV overpotential at 1 mA cm^−2^)^15^, electrodeposited H_2_-CoCat catalysts (385 mV overpotential at 2 mA cm^−2^)^39^, ordered double-gyroid MoS_2_ bicontinuous networks, and chemically exfoliated WS_2_ nanosheets (∼240 mV overpotential at 10 mA cm^−2^ for the latter two cases)^21^,^23^. In addition, the overpotential reached by the CoN_*x*_/C catalysts is comparable to those of highly active metal phosphide and carbide nanoparticle catalysts (70–130 mV overpotential at 10 mA cm^−2^)^25^,^27^.

The electrochemical impedance spectroscopy (EIS) analysis also confirmed a faster HER kinetic process on CoN_*x*_/C than on N/C and Co/C (Supplementary Fig. 5). Furthermore, we found that the HER activity of CoN_*x*_/C continuously increased with the cobalt/*o*PD ratio (up to ∼10 wt%) in the precursor materials (Supplementary Fig. 6). Further increasing the cobalt/*o*PD ratio to 20 wt% caused a dramatic decrease in activity, primarily due to the seriously reduced porosity of the carbon-based catalysts. The strong correlation between the HER activity and the amount of metal in the 0–10 wt% range probably indicates that the cobalt species are directly related to the active sites (see below for more details of the discussion).

The evolution of hydrogen on different catalysts was confirmed by the rotating ring disk electrode (RRDE) technique^14^. The disk electrode bearing the catalyst was rotated at 1,600 r.p.m. to remove any H_2_ produced on the disk radially. Simultaneously, the Pt ring that encircles the disk electrode was maintained at 0.7 V versus reversible hydrogen electrode (RHE) to oxidize the H_2_ and produce a ring current. The selective detection of H_2_ formation by RRDE confirmed that all the catalysts generated H_2_ within the defined potentials (Fig. 2b). In particular, the electrocatalytic hydrogen evolution on the CoN_*x*_/C-modified electrode occurred with an overpotential of only 20 mV (Fig. 2b), much lower than that observed on N/C- (∼220 mV) and Co/C- (∼60 mV) modified electrodes.

Figure 2c displays the Tafel plots of the polarization curves that provide insight into the HER pathways on various catalysts. The Pt/C-modified electrode exhibited a Tafel slope of ∼30 mV per decade, which is consistent with the known mechanism of HER on Pt. The N/C and Co/C catalysts showed a Tafel slope of ∼98 and ∼106 mV per decade, respectively, suggesting that an initial proton adsorption was the rate-determining step on these two catalysts^40^. The Tafel analysis of the CoN_*x*_/C catalyst revealed a Tafel slope of ∼57 mV per decade, which may suggest hydrogen production via the Volmer–Heyrovsky mechanism, and that the electrochemical desorption step is rate limiting^40^. On the basis of the Tafel analysis, the exchange current density of the CoN_*x*_/C catalyst was estimated to be ∼7 × 10^−5^ A cm^−2^. This value is much higher than that reported for the electrodeposited H_2_-CoCat catalyst (∼3.2 × 10^−6^ A cm^−2^)^39^, CNTs-supported nickel bisdiphosphine complex (∼3.2 × 10^−7^ A cm^−2^)^14^, and C_3_N_4_/N-doped graphene (3.5 × 10^−7^ A cm^−2^)^41^.

Electrochemical stability is an important requirement for a HER catalyst. An accelerated degradation study^25^,^42^ was therefore carried out to assess the stability of the CoN_*x*_/C catalyst in 0.5 M H_2_SO_4_. For comparison, the Co/C catalyst was also examined under the same condition. Although the Co/C catalyst showed a moderate activity at the beginning, the HER performance rapidly decreased with the potential cycling (Fig. 2d). After 100 cyclic voltammetry (CV) cycles, the overpotential required to achieve a current density of 10 mA cm^−2^ on the Co/C catalyst increased by >40 mV. The rapid degradation of the Co/C catalyst is likely associated with the gradual leaching out of cobalt species during the electrochemical process in the strong acid electrolyte. In sharp contrast, even after 5,000 CV cycles, only a slight negative shift of the HER polarization of the CoN_*x*_/C catalyst (11 mV shift of the overpotential at 10 mA cm^−2^) was observed, which suggests the superior durability of CoN_*x*_/C for HER.

Encouraged by the high electrochemical stability of the CoN_*x*_/C catalyst, we further studied the HER performance in alkaline and neutral media. The CoN_*x*_/C system catalysed the HER much more efficiently than did the N/C and Co/C catalysts in both the alkaline and neutral media, exhibiting an overpotential of 170 and 247 mV at a current density of 10 mA cm^−2^ in 1.0 M KOH and 1.0 M phosphate buffer solutions (pH 7), respectively (Fig. 2e and Supplementary Fig. 7). These overpotentials compare favourably with the values reported for most non-precious metal HER catalysts under similar conditions (Supplementary Table 2), such as cobalt nanoparticle-based catalysts^34^,^43^, tungsten carbonitride^31^ and copper molybdenum sulfide^44^. The CoN_*x*_/C catalyst showed a Tafel slope of ∼75 mV per decade in alkaline medium, which is much lower than those of N/C and Co/C catalysts (Fig. 2f). Moreover, the CoN_*x*_/C catalyst also exhibited negligible degradation in both alkaline and neutral media (Supplementary Fig. 8). The ability to operate over a wide range of pH makes the CoN_*x*_/C catalyst very flexible to coupling with favourable anodic catalysts for different types of water splitting devices^1^,^45^.

### Physical characterization of the CoN_*x*_/C catalysts

**Figure 3:**
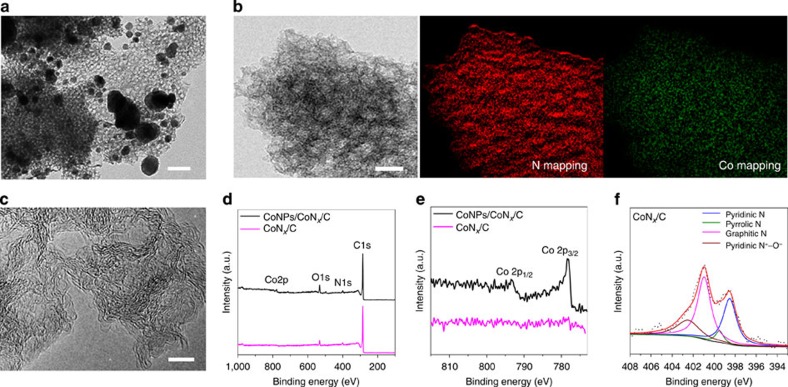
Catalyst characterization. (**a**) TEM image of CoNPs/CoN_*x*_/C. Scale bar, 100 nm. (**b**) TEM image of CoN_*x*_/C and corresponding EFTEM elemental mapping demonstrating the homogeneous distribution of both cobalt and nitrogen at the atomic scale. Scale bar, 20 nm. (**c**) High-resolution TEM image of CoN_*x*_/C showing the layered graphene structure without any metal particles or nanoclusters. Scale bar, 5 nm. (**d**) XPS survey spectra of CoNPs/CoN_*x*_/C and CoN_*x*_/C. (**e**) High-resolution Co2p spectra of CoNPs/CoN_*x*_/C and CoN_*x*_/C. (**f**) High-resolution N1s spectra of CoN_*x*_/C.

To gain insights into the origin of the superior catalytic activity of the CoN_*x*_/C catalyst, different physical characterizations were conducted. Transmission electron microscopy (TEM) observations revealed that the CoNPs/CoN_*x*_/C catalyst prepared without acid leaching consisted of mesoporous carbon frameworks dispersed with irregular inorganic particles (Fig. 3a) that were identified as metallic cobalt by X-ray diffraction analysis (Supplementary Fig. 9). Upon leaching with acid, all cobalt nanoparticles were removed, as indicated by the TEM image and X-ray diffraction patterns (Fig. 3b and Supplementary Fig. 9, 10) of the CoN_*x*_/C catalyst. The absence of cobalt nanoparticles in the CoN_*x*_/C catalyst was further confirmed by magnetic measurement (Supplementary Fig. 11). N_2_ sorption tests were performed to evaluate the porous properties of the catalysts (Supplementary Figs 12, 13 and Supplementary Table 3). The steep and high capillary condensation steps in the isotherms revealed a uniform and well-developed mesoporosity with a large mesopore volume, which is in good agreement with the TEM observations. The considerable adsorption at the low relative pressure indicated the microporosity as well. Notably, the acid leaching resulted in a considerable increase in the apparent Brunauer–Emmett–Teller (BET) surface area from 745 m^2^ g^−1^ for CoNPs/CoN_*x*_/C to 1,074 m^2^ g^−1^ for CoN_*x*_/C without deterioration of the mesoporosity.

The high-resolution TEM image disclosed that the CoN_*x*_/C catalyst is composed of randomly orientated graphene layers without any metal particle or nanoscaled clusters (Fig. 3c). Importantly, elemental mapping with sub-nanoscale energy-filtered TEM (EFTEM) imaging revealed that both the nitrogen and the cobalt elements were homogeneously distributed throughout the whole carbon matrix (Fig. 3b). X-ray photoelectron spectroscopy (XPS) analysis was performed to probe the chemical composition of CoNPs/CoN_*x*_/C and CoN_*x*_/C. The surface nitrogen and cobalt contents of the CoNPs/CoN_*x*_/C catalyst were 3.3 (±0.3) and 1.2 (±0.3) at%, respectively (Fig. 3d and Supplementary Table 3). After acid leaching, the nitrogen content increased slightly to 3.9 (±0.3) at% for the CoN_*x*_/C catalyst, but no cobalt signal could be observed because of the relatively high detection limit (∼0.1 at%) of the XPS technique (Fig. 3e). Inductively coupled plasma atomic emission spectrometry (ICP-AES) showed a bulk cobalt content of 0.14 (±0.02) wt% in the CoN_*x*_/C catalysts, significantly less than that of the CoNPs/CoN_*x*_/C catalyst (7.5±0.5 wt%). The high-resolution N1s spectrum suggested that the CoN_*x*_/C catalyst contained primarily pyridinic and graphitic nitrogen, together with a small amount of pyrrolic nitrogen and oxidized nitrogen species (Fig. 3f).

### Understanding the active sites

**Figure 4:**
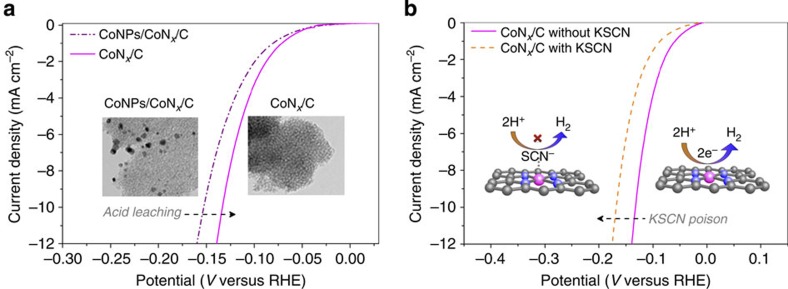
Understanding the structure of the active sites. (**a**) Comparison of the HER activity of the CoNPs/CoN_*x*_/C and CoN_*x*_/C catalysts showing the influence of acid leaching. Insets are TEM images demonstrating that all cobalt particles were removed by acid leaching. (**b**) HER polarization plots of CoN_*x*_/C with and without 10 mM KSCN in 0.5 M H_2_SO_4_, indicating that SCN^−^ ions strongly poison the CoN_*x*_/C catalyst. Insets are illustrations of cobalt centres blocked by the SCN^−^ ions. These measurements indicated that the cobalt is involved in the active centers but not in the form of metallic nanoparticles.

The influence of the acid leaching of the CoN_*x*_/C catalyst on the HER activity was studied to determine whether the metallic cobalt nanoparticles contribute to the electrocatalytic HER. By comparing the HER polarization curves of the CoNPs/CoN_*x*_/C and CoN_*x*_/C catalysts, we found a sufficient improvement in HER activity after acid leaching (Fig. 4a). Such observation clearly excludes the contribution of metallic cobalt or cobalt oxide nanoparticles to the HER that was recently reported to be at least partly responsible for the catalytic hydrogen evolution^34^,^35^,^39^,^46^. In our catalyst system, acid leaching eliminated the inactive inorganic cobalt phase and exposed a larger carbon surface, which resulted in an increased BET surface area, as confirmed by the N_2_ sorption tests (Supplementary Fig. 12), which in turn led to enhanced HER performance.

To further understand the nature of the active sites of the CoN_*x*_/C catalyst, we investigated the influence of thiocyanate ions (SCN^−^) on the HER activity of the CoN_*x*_/C, N/C and Co/N catalysts. SCN^−^ is widely known to poison the metal-centred catalytic sites in acidic conditions^47^,^48^. On introducing SCN^−^ into the acidic electrolyte (10 mM), the overpotential of the CoN_*x*_/C catalyst increased by >35 mV (Fig. 4b) and the current density significantly decreased from 16.2 to 6.2 mA cm^−2^ at the overpotential of 150 mV (Supplementary Fig. 14), which suggests that >60% cobalt sites were blocked by the SCN^−^ ions. Interestingly, no appreciable decrease of HER activity was observed for the N/C catalyst in the presence of SCN^−^ ions (Supplementary Fig. 15), which indicated that there were no poisoning effects of SCN^−^ on the metal-free nitrogen-doped carbon catalyst. Additionally, the SCN^−^ ions also poisoned seriously the nitrogen-free Co/C catalyst (Supplementary Fig. 15), although the cobalt-centred sites without nitrogen coordination were instable in acidic conditions, as revealed by the accelerated degradation study (Fig. 2d). These results, combined with the EFTEM mapping as well as with XPS and high-resolution TEM analyses, strongly highlighted that after acid leaching, the surviving ionic cobalt species were well distributed in the carbon/nitrogen matrix and coordinated with nitrogen, at least partly, to form active and acid-resistant CoN_*x*_ centres.

### TOF of the CoN_*x*_/C catalyst

**Figure 5:**
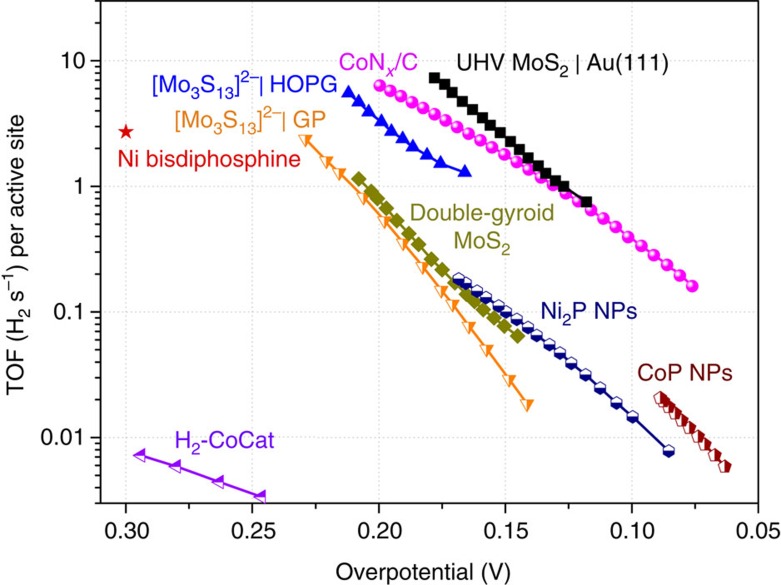
Comparison of the TOF of CoN_*x*_/C with other catalysts. TOF plots of the CoN_*x*_/C catalyst together with other recently reported molecular and inorganic HER catalysts. Data adapted from: ref. 17 for UHV MoS_2_|Au (111); ref. 42 for [Mo_3_S_13_]^2−^|HOPG and [Mo_3_S_13_]^2−^|graphite paper; ref. 21 for double-gyroid MoS_2_; ref. 25 for Ni_2_P NPs; ref. 27 for CoP NPs; ref. 14 for Ni-bisdiphosphine/CNTs; ref. 39 for H_2_-CoCat. Data for CoN_*x*_/C is from the present study.

After assigning the active sites of the CoN_*x*_/C catalyst to the cobalt-containing centres, we estimated the TOF per cobalt site, which indicates the intrinsic per-site activity of a catalyst and is the best figure of merit for comparing activities among different catalysts. We determined the upper limit of the active sites to be ∼2.85 × 10^16^ sites per cm^2^, based on the hypothesis that all the cobalt atoms in the CoN_*x*_/C catalyst formed active CoN_*x*_ centres and all of them were accessible to the electrolyte (details of the calculation are provided in the Supplementary Information and Supplementary Note 1). Accordingly, the TOF values per cobalt site of the CoN_*x*_/C catalyst were calculated by this method and plotted against the applied overpotential, as shown in Fig. 5. In particular, the TOFs of CoN_*x*_/C are 0.39 H_2_ per s and 6.5 H_2_ per s at overpotentials of 100 and 200 mV, respectively. For the purpose of direct comparison, Fig. 5 also includes the TOF values of seven state-of-the-art non-precious metal HER catalysts published recently, including UHV-deposited MoS_2_ nanoparticles supported on Au(111) (ref. 17), [Mo_3_S_13_]^2−^ clusters on HOPG and graphite paper^42^, double-gyroid MoS_2_ (ref. 21), Ni_2_P NPs (ref. 25), CoP NPs (ref. 27), Ni-bisdiphosphine molecular catalysts supported on CNTs (ref. 14), and electrodeposited H_2_-CoCat catalysts^39^. Clearly, the CoN_*x*_/C catalyst outperformed the recently reported scalable molecular or solid-state HER catalysts. The TOF values of CoN_*x*_/C are only slightly lower than those of UHV-deposited MoS_2_ nanoparticles at overpotentials larger than 120 mV, although the latter could not be prepared via a scalable synthesis route^17^.

## Discussion

To summarize, we demonstrated scalable, solid-state, carbon-based HER catalysts that contain molecular CoN_*x*_ active centres and exhibit an extremely high activity and excellent stability in aqueous solutions over a wide range of pH values. We attribute the outstanding HER performance of the CoN_*x*_/C catalysts to the CoN_*x*_ centres generated during the pyrolysis of the metal–N_4_ macrocycles or cobalt-*o*-phenylenediamine complexes, which are identified to be the active sites for HER by the acid leaching and thiocyanate ion poisoning experiments. The TOF values per cobalt site towards HER of our catalysts in an acidic electrolyte are substantially higher than those of reported non-precious metal molecular and solid-state HER catalysts. Further mechanistic studies are still required to unravel the exact structure of the active sites of the CoN_*x*_/C catalyst and to better understand the involved reaction mechanism.

There is still a large room for the further improvement of the HER activity of MeN_*x*_/C catalysts. Optimizing the Co–N binding properties or increasing the porosity by regulating the synthetic parameters (for example, NH_3_ post treatment^37^,^49^) would be an effective way to enhance the intrinsic activity per site as well as the HER performance on a geometric area basis. Further exploration of other MeN_*x*_ or bimetallic centres for HER would also provide many opportunities in this field, although we found that CoN_*x*_ was the best among several MeN_*x*_/C catalysts (Supplementary Fig. 16). Moreover, bimetallic centres (for example, CoNi–N_*x*_, CoMn–N_*x*_) or even other coordinated atoms (for example, molecular Me–S_*x*_ (ref. 42) and Me–P_*x*_ (ref. 13) centres) could potentially lead to an enhancement in the catalytic activity for the HER and finally enable the catalysts to compete against the best Pt/C catalyst in technological devices. Considering the much lower metal content of the CoN_*x*_/C catalyst than those of the recently reported metal nanoparticle-based catalysts^34^,^35^,^43^, future work will be also dedicated to increasing the density of CoN_*x*_ sites. The heterogeneous solid-state catalysts with molecular active sites described in the present work shed new light on the future development of earth-abundant catalysts for other important electrochemical processes, such as oxygen evolution, hydrogen oxidation and CO_2_ reduction.

## Methods

### Electrocatalyst synthesis

Three different precursors have been used for the synthesis of the CoN_*x*_/C catalysts, including a mixture of *o*PD and Co(NO_3_)_2_, CoTMPP and VB12 (Fig. 1). To prepare the CoN_*x*_/C (Co-*o*PD) catalyst, 3.0 g *o*PD monomer was dissolved in 60 ml 1.0 M HNO_3_; then, 22.5 g silica colloid solution (Ludox HS40, ∼12 nm, 40 wt%) and 1.5 g Co(NO_3_)_2_·6H_2_O were added. After stirring for ∼10 min, the mixture was dried by using a rotary evaporator. The dried powder was pyrolyzed under flowing N_2_ for 2 h at temperatures ranging from 700 to 1,000 °C. Then, the silica template was etched out with 2.0 M NaOH. Subsequently, the sample was subjected to the second heat treatment in N_2_ atmosphere for 2 h to form the CoNPs/CoN_*x*_/C composite. Finally, the catalyst was leached in 0.5 M H_2_SO_4_ at 90 °C for 4 h to remove the cobalt-containing particles and was heat-treated again in N_2_ atmosphere for 2 h. All heat treatments were performed at the same temperature.

For comparison, a metal-free N-doped carbon catalyst (N/C) was prepared by the same process, although without adding Co(NO_3_)_2_·6H_2_O. The nitrogen-free Co-doped catalyst (Co/N) was prepared by the pyrolysis of a mixture containing 3.0 g sucrose, 15 g Ludox HS40 silica colloid (dry weight, 6.0 g), and 1.5 g Co(NO_3_)_2_·6H_2_O, followed by the same heat treatment and NaOH/H_2_SO_4_ etching steps. A pure carbon catalyst was prepared by the same method although without adding Co(NO_3_)_2_·6H_2_O.

For the synthesis of the CoN_*x*_/C (CoTMPP) catalyst, a mixture of 1.0 g CoTMPP and 1.0 g fumed silica (7 nm, Sigma-Aldrich S5130) was thoroughly grinded in an agate mortar for at least 30 min. Then, the mixed powder was subjected to the heat treatment and NaOH/H_2_SO_4_ etching steps. For the synthesis of the CoN_*x*_/C (VB12) catalyst, 1.0 g VB12 was first dissolved in 50 ml distilled water, and 2.5 g silica colloid solution (Ludox HS40, ∼12 nm, 40 wt%) was then added. After drying the solution with a rotary evaporator, the mixture underwent the same heat treatment and NaOH/H_2_SO_4_ etching process, as described above.

For each CoN_*x*_/C catalyst, the temperature of the heat treatment was optimized as a function of HER activity. The best temperature was found to be 900, 800 and 700 °C for the Co-*o*PD, CoTMPP and VB12-derived catalysts, respectively (Supplementary Fig. 1).

### Electrochemical measurements

All electrochemical measurements were carried out in a conventional three-electrode cell using a WaveDriver 20 bipotentiostat (Pine Instrument Company, USA) controlled at room temperature. Ag/AgCl (4 M KCl) and platinum wire were used as reference and counter electrodes, respectively. All potentials in this study refer to those of the RHE. The potential difference between Ag/AgCl and RHE was determined based on the calibration measurement in H_2_-saturated electrolyte. An RDE with a glassy carbon disk (5.0 mm diameter) and an RRDE with both a Pt ring (6.25 mm inner-diameter and 7.92 mm outer-diameter) and a glassy carbon disk (5.61 mm diameter) served as the substrate for the working electrodes in evaluating the HER activity and confirming the hydrogen evolutions. Before use, the glassy carbon electrodes in RDE/RRDE were polished using aqueous alumina suspensions on felt polishing pads. For the stability study, a graphite rod was used as the counter electrode to avoid the possible contribution of dissolved Pt species to the HER.

The catalyst ink was prepared by blending 10 mg of each catalyst with 100 μl Nafion solution (0.5 wt%) and 0.4 ml ethanol in an ultrasonic bath. A certain volume of catalyst ink was then pipetted onto the glassy carbon surface to result in the desirable catalyst loading. The RDE/RRDE tests were measured in an Ar-saturated electrolyte at 1,600 r.p.m. with a sweep rate of 5 mV s^−1^. The measured HER polarization curves were capacity corrected by taking an average of forward and backward (positive and negative-going) scans. For detecting the hydrogen evolved at the disc electrode, the potential of the Pt-ring electrode in the RRDE system was set to 0.7 V versus RHE. EIS spectra were recorded in an Ar-saturated electrolyte with a 5 mV AC potential from 10 kHz to 0.01 Hz at 1,600 r.p.m. For comparison, the commercial 20 wt% platinum on Vulcan carbon black catalyst (Pt/C from BASF) was measured under the same experimental conditions. The catalysts loading are 2.0 mg cm^−2^ for non-Pt materials and 0.2 mg cm^−2^ (40 μg_Pt_ cm^−2^) for the Pt/C catalyst. For the CoN_*x*_/C catalyst, the cobalt loading is only 2.8 μg_Co_ cm^−2^, based on the ICP-AES measurements.

### Characterization

TEM was performed using a JEM-1400 microscope (JEOL Ltd., Japan) operating at an accelerating voltage of 200 kV. High-resolution TEM measurements were conducted on a Tecnai F20 (FEI) microscope with a beam voltage of 200 kV. EFTEM mapping were performed using a JEM-ARM 200F microscope operating at an accelerating voltage of 200 kV. Elemental mapping were collected using a Gatan GIF Quantum 965. XPS experiments were carried out on an AXIS Ultra DLD system from Kratos with Al Kα radiation. X-ray diffraction spectra were recorded on a PW1820 powder diffractometer (Phillips) using a Cu-Kα emitter. Nitrogen sorption measurements were conducted at 77 K on a TriStar 3020 volumetric analyser (Micromeritics). All samples were degassed at 300 °C for at least 4 h before every measurement. Specific surface areas were determined by the standard BET method based on the relative pressure between 0.05 and 0.20. The pore size distribution was calculated using the non-local density functional theory method. ICP-AES measurements were conducted on an Atomscan Advantage Spectrometer (Thermo Ash Jarrell Corporation).

## Additional information

**How to cite this article:** Liang, H.-W. *et al*. Molecular metal–N_*x*_ centres in porous carbon for electrocatalytic hydrogen evolution. *Nat. Commun.* 6:7992 doi: 10.1038/ncomms8992 (2015).

## Supplementary Material

Supplementary Information Supplementary Figures 1-16, Supplementary Table 1-3, Supplementary Note 1 and Supplementary References
