# Supplementary material for: Molecular metal–Nx centres in porous carbon for electrocatalytic hydrogen evolution
Source: Nat Commun. 2015 Aug 7;6:7992. doi: 10.1038/ncomms8992 (PMC4918366; doi:10.1038/ncomms8992)
Supplement: Supplementary Information — Supplementary Figures 1-16, Supplementary Table 1-3, Supplementary Note 1 and Supplementary References [file ncomms8992-s1.pdf]

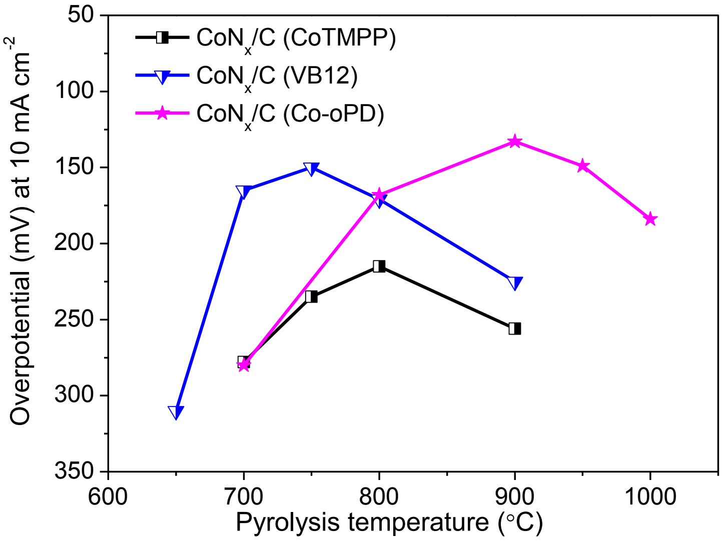

Supplementary Figure 1. HER activity of CoN_x/C catalysts prepared with different precursors as a function of pyrolysis temperature. Three precursors have been used in this work, including metal-N_4 macrocycles (CoTMPP), biomolecules (VB12), and a mixture of cobalt salts and nitrogen-containing aromatic molecules (Co-*o*PD). The pyrolysis temperature was separately optimized from 600 to 900 °C for each precursor. The best pyrolysis temperature was found to be 800, 750, and 900 °C for CoTMPP, VB12, and Co-*o*PD, respectively. The VB12-derived carbon catalyst exhibited a slightly lower HER activity than the Co-*o*PD-derived catalyst but a significantly higher HER activity than the CoTPMM-derived catalyst.

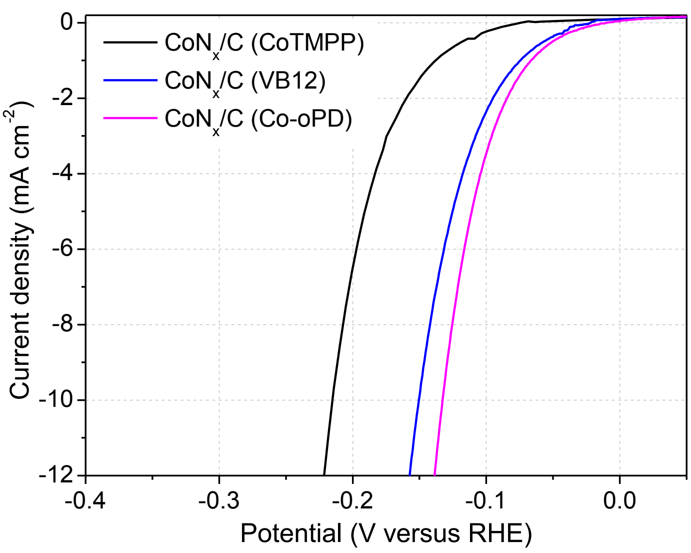

Supplementary Figure 2. Comparison of the HER activity of CoN_x/C catalysts prepared with different precursors. Each catalyst has been optimized as function of pyrolysis temperature, as shown in Figure S1. The VB12-derived carbon catalyst exhibited a slightly lower HER activity than the Co-*o*PD-derived catalyst but a significantly higher HER activity than the CoTPMM-derived catalyst.

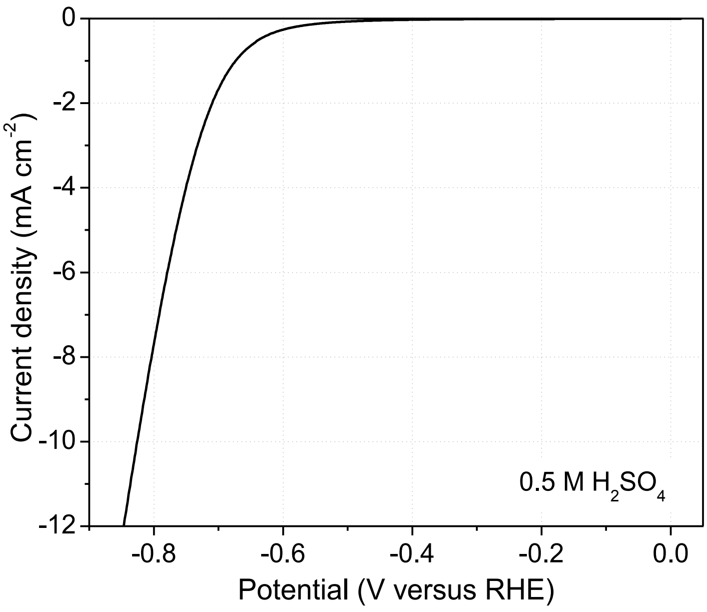

Supplementary Figure 3. HER polarization curve of non-doped porous carbon prepared with sucrose as precursor and silica colloid as template.

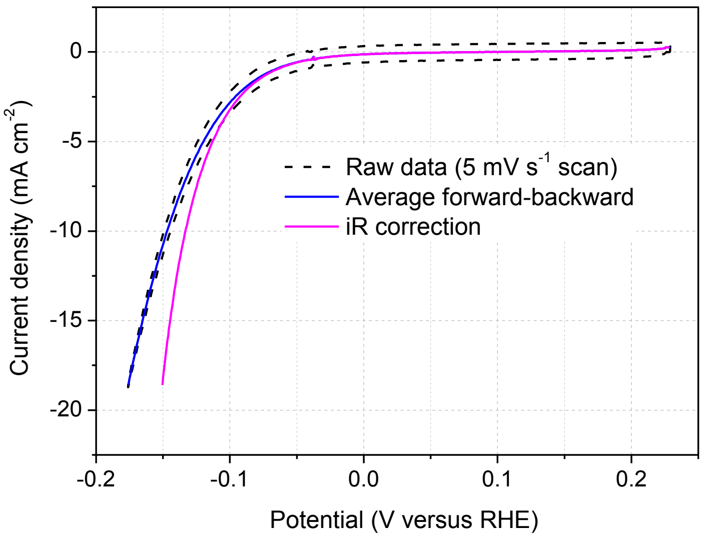

Supplementary Figure 4. Ohmic and capacitive corrections of the as-measured HER activity of the CoN_x/C catalyst. The as-measured HER activity of the CoN_x/C catalyst ("raw", dashed black line) is capacity-corrected by taking an average of forward and backward (positive and negative-going) scans. The capacity-corrected HER current (solid blue line) is then ohmically corrected with the measured ionic resistance ($\approx 7\ \Omega$) to yield the final electrode HER activity (solid magenta line).

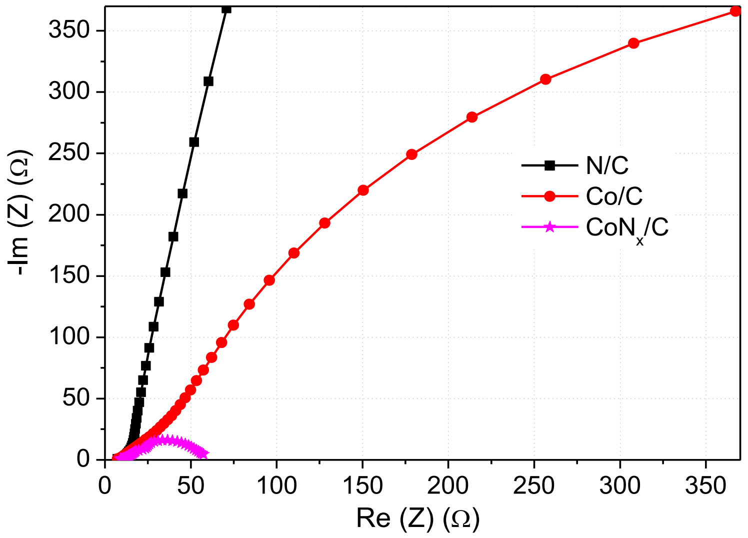

Supplementary Figure 5. Electrochemical impedance spectroscopy (EIS) analyses of the catalysts. EIS measurements were recorded on RDE in Ar-saturated 0.5 M H_2SO_4 at –0.1 V vs. RHE with 5 mV AC potential from 10 kHz to 0.01 Hz. Electrode rotation speed: 1600 rpm. The measured impedance can be presented in the form of imaginary (Im) vs. real (Re) parts at various frequencies. It has been recognized that the high frequency interception of the Re-axis represents the resistance of the electrodes and that the width of the semicircle on the Re-axis corresponds to the charge-transfer resistances and indicates the overall kinetic effects. Clearly, all three catalysts exhibited almost the the same intrinsic resistance, while the charge-transfer resistance of CoN_x/C was much lower than that of N/C and Co/C, suggesting a faster HER kinetic process on CoN_x/C, which is in good agreement with the RDE analysis.

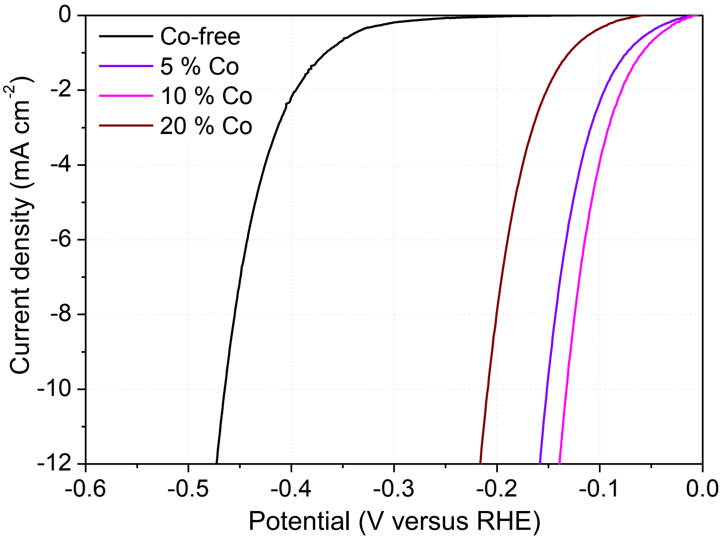

Supplementary Figure 6. Comparison of the HER activity of the N/C and CoN_x/C catalysts prepared with increasing Co/*o*PD ratios from 5.0 to 20 wt%. The strong positive shift of the HER polarization curves indicated that the incorporation of only 5 wt% Co could dramatically improve the HER performance compared that of the metal-free N/C catalyst. The optimal Co/*o*PD ratio was found to be 10 wt%. Further increasing the ratio from 10 to 20 wt% resulted in a much lower HER activity due to the greatly reduced specific surface area (S_{BET} = 1070 m^2 g^{-1} for 10 wt% vs. 410 m^2 g^{-1} for 20 wt%).

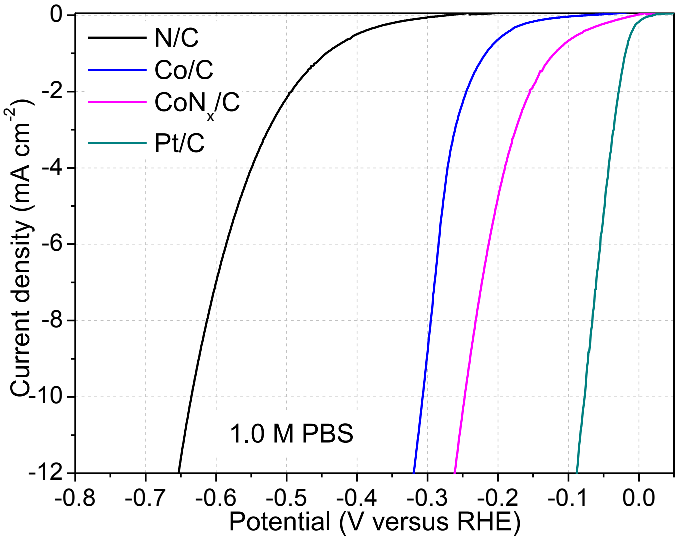

Supplementary Figure 7. HER activity of the catalysts in a neutral medium. HER polarization curves of CoN_x/C and reference materials in 1.0 M PBS, indicating that the CoN_x/C catalyst can also work very well in neutral media, with an overpotential of 247 mV at a current density of 10 mA cm^{-2}.

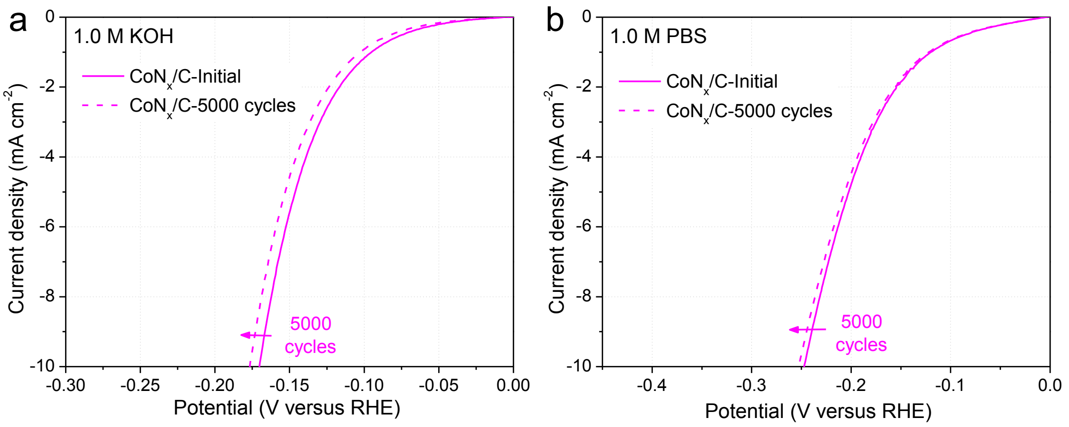

Supplementary Figure 8. HER polarization of CoN_x/C initially and after 5000 cyclic voltammograms cycles between 0.20 V and −0.25 V vs. RHE (not iR-corrected) in 1.0 M KOH (a) and 1.0 M PBS (b).

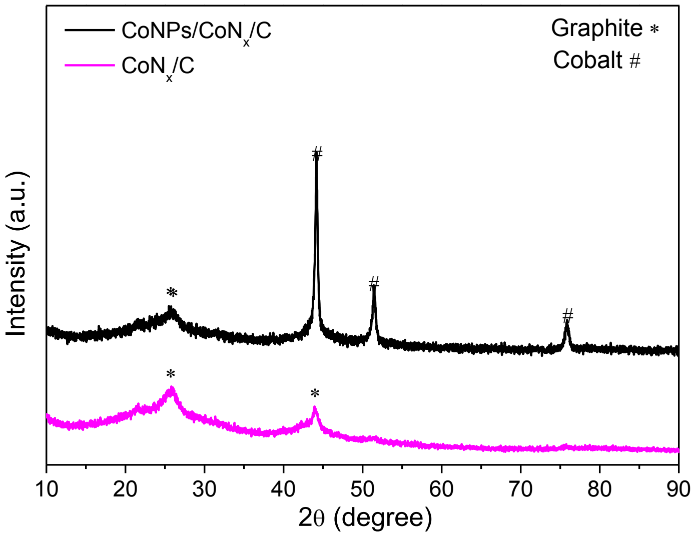

Supplementary Figure 9. XRD patterns of CoNPs/CoN_x/C and CoN_x/C, further conforming the absence of any other inorganic crystalline phase in leached sample.

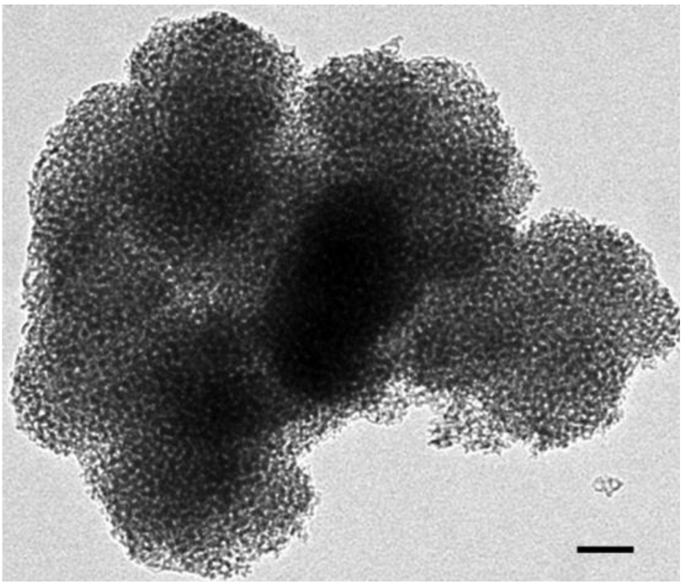

Supplementary Figure 10. Additional TEM image of the CoN_x/C catalyst. Scale bar: 100 nm. All cobalt-containing nanoparticles were leached out by acid.

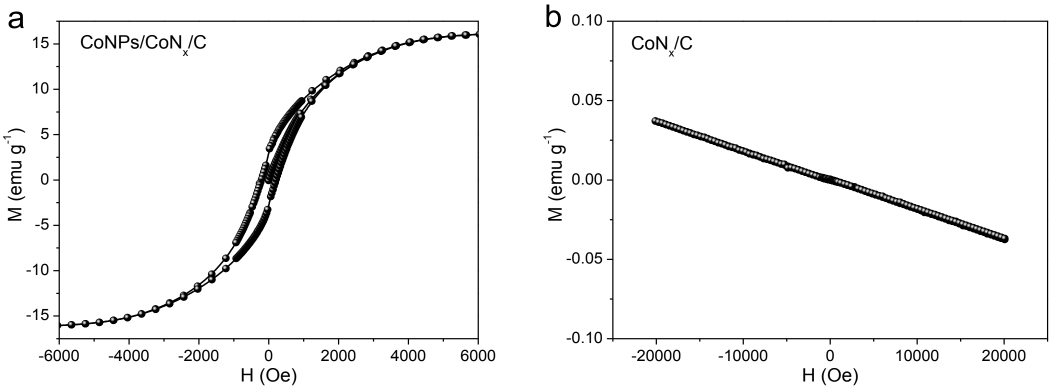

Supplementary Figure 11. Room-temperature magnetic hysteresis loops at 300 K of (a) $CoNPs/CoN_x/C$ and (b) CoN_x/C catalysts. While the $CoNPs/CoN_x/C$ exhibited a strong metallic ferromagnetic behavior, the CoN_x/C catalyst showed diamagnetic state, which the typical magnetic property of carbons. The magnetic data further confirmed that all metallic cobalt nanoparticles have been leached out by acid treatment.

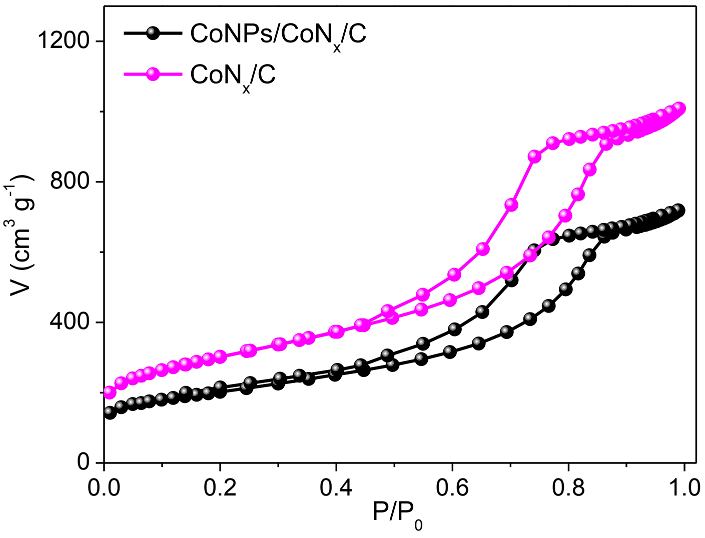

Supplementary Figure 12. N_2 adsorption/desorption isotherms of CoNPs/CoN$_x$/C and CoN$_x$/C, showing an increasing BET surface area after the leaching out of cobalt nanoparticles.

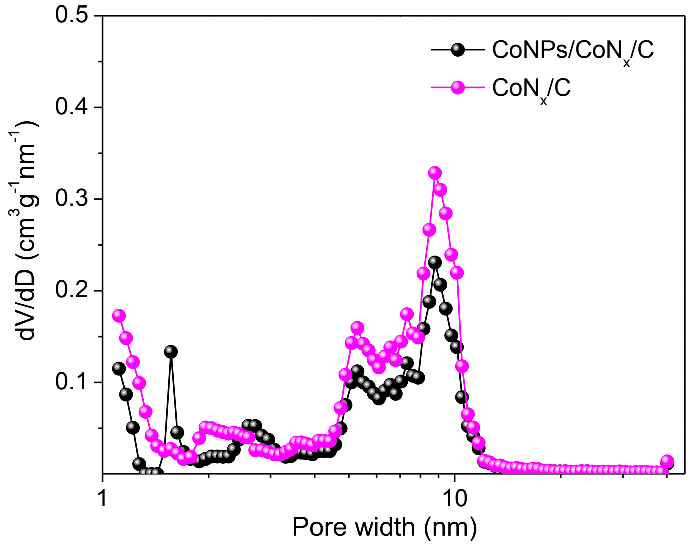

Supplementary Figure 13. Pore size distribution of CoNPs/CoN$_x$/C and CoN$_x$/C catalysts based on calculation with the NLDFT method.

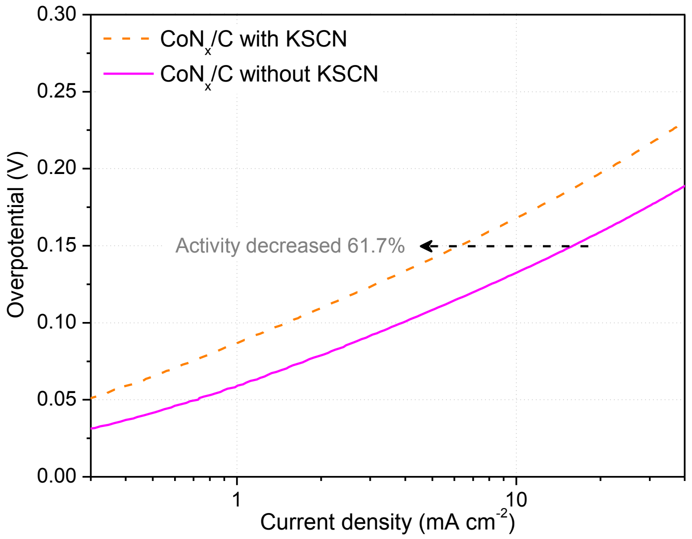

Supplementary Figure 14. Tafel plots obtained from the HER polarization curves in Figure 4b, indicating that the activity of CoN_x/C catalyst at the overpotential of 150 mV decreased 61.7% upon introducing SCN^- into the electrolyte.

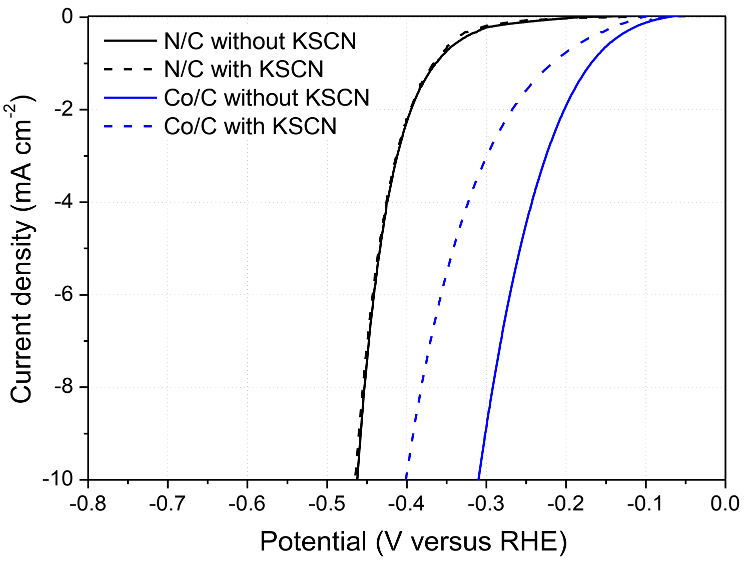

Supplementary Figure 15. KSCN poisoning of the N/C and Co/C catalysts. HER polarization curves of N/C and Co/C catalysts with and without 10 mM KSCN in Ar-saturated 0.5 M H_2SO_4. Similarly to the CoN_x/C catalyst, the polarization curve of the Co/C catalyst is greatly negatively shifted upon addition of 10 mM KSCN into the acidic electrolyte, indicating a direct contribution of Co to the HER. In contrast, we did not observe any obvious effect of the SCN^- ions on the HER performance of metal-free N/C catalysts.

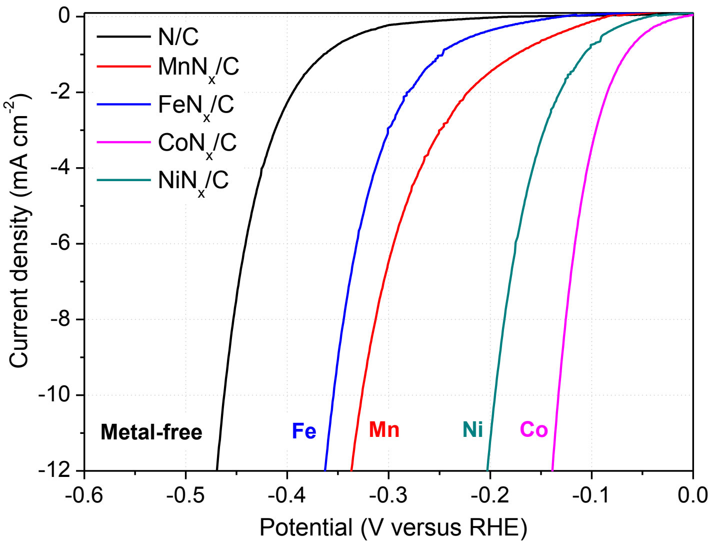

Supplementary Figure 16. Comparison of the HER activity of MeN_x/C catalysts with different metal centers in 0.5 M H_2SO_4. The order of HER activity is Co > Ni >> Mn > Fe >> metal-free.

Supplementary Table 1. Summary of recently reported HER performance of molecular as well as solid-state catalysts in acidic aqueous solution.

Catalyst	Catalyst loading (mg cm^{-2})	Onset overpotential (mV)	Overpotential at10 mA cm^{-2} (mV)	TOF (H_2 s^{-1})	Tafel slope (mV/decade)	Reference
Ni-bisdiphosphine/CNTs	0.06	20	>300	2.78 at 300 mV overpotential	unknown	1
Cobalt diimine-dioxime complexes	unknown	350	>590	2.2 at 590 mV overpotential	160	2
Electrodeposited H_2-CoCat	0.059 (only Co)	50	>385	0.02 at 380 mV overpotential	140	3
UHV MoS_2|Au (111)	<monolayer	~100	unknown	2.4 at 150 mV overpotential	55-60	4
$[Mo_3S_{13}]^{2-}$|GP	0.1	100	180	0.59 at 200 mV overpotential	38-40	5
Amorphous molybdenum sulfide	unknown	~150	>200	0.8 at 220 mV overpotential	40	6
Double-gyroid MoS_2	unknown	150	240	0.78 at 200 mV overpotential	50	7
MoS_2 Nanosheets	unknown	~150	187	unknown	43	8
Ni_2P nanoparticles	1.0	~50	187	0.015 at 100 mV overpotential	46	9
CoP nanoparticles	2.0	~0	75	0.014 at 80 mV overpotential	50	10
$NiMoN_x$ nanosheets/C	0.25	78	>200	unknown	35.9	11
Mo_2C/CNTs	2.0	~0	152	unknown	55	12
Tungsten Carbonitride	0.4	100	220	unknown	47	13
C_3N_4@NG	0.1	~150	240	unknown	51.5	14
Porous C_3N_4@N-G films	unknown	8	80	unknown	49.1	15
Co NPs@N-CNTs	0.28	50	260	unknown	69	16
FeCo NPs@N-CNTs	0.32	70	280	unknown	74	16
Fe NPs@single shell carbon	0.18	0	77	unknown	40	17
**CoN_x/C**	**2.0**	**20**	**133**	**0.39/6.5 at 100/200 mV overpotential**	**57**	**This work**

Supplementary Table 2. Summary of HER performance of reported catalysts in alkaline and neutral aqueous solution.

Catalyst	Electrolyte	Catalyst loading (mg cm^{-2})	Onset overpotential (mV)	Overpotential at10 mA cm^{-2} (mV)	Reference
Mo_2C	1.0 M KOH	0.8	110	190	18
Ni-Mo nanopowders	1.0 M NaOH	1.0	~0	80	19
Tungsten Carbonitride	unknown (pH = 13)	0.4	120	250	13
Co NPs@N-CNTs	1.0 M KOH	0.28	~100	370	16
Co NPs@N-CNTs	1.0 M PBS (pH = 7)	0.28	~250	540	16
NiO/Ni-CNT	1.0 M KOH	0.28	~0	80	20
Cu_2MoS_4	0.1 M PBS (pH = 7)	0.04	~550	~750	21
Co NPs@N-C	1.0 M KOH	unknown	~50	210	22
**CoN_x/C**	**1.0 M KOH**	**2.0**	**30**	**170**	**This work**
**CoN_x/C**	**1.0 M PBS (pH = 7)**	**2.0**	**30**	**247**	**This work**

Supplementary Table 3. Elemental composition measured by XPS and ICP-AES and specific surface area of the CONPs/CoN_x/C and CoN_x/C catalysts.

sample	Co-XPS (at%)	Co-ICP (wt%)	N-XPS (at%)	O-XPS (at%)	C-XPS (at%)	S_{BET} (m^2 g^{-1})
CoNPs/CoN_x/C	1.16	7.7	2.96	6.7	89.18	745
CoN_x/C	ND	0.14	3.86	4.52	91.18	1070

ND, no Co was detected by XPS.

Supplementary Note 1. TOF calculation of the CoN_x/C catalyst.

To calculate the turnover frequency (TOF) per cobalt site in the CoN_x/C catalyst, we used the following formula:

$$TOF = \frac{\#\ \text{total hygrogen turnover} \ / \ \text{cm}^2 \ \text{geometric aera}}{\#\ \text{active sites} \ / \ \text{cm}^2 \ \text{geometrica rea}} \tag{1}$$

The total number of hydrogen turn overs was calculated from the current density according to[5,23]:

$$\#_{H_2} = \left(j \frac{\text{mA}}{\text{cm}^2} \right) \left(\frac{1\text{C/s}}{1000\text{mA}} \right) \left(\frac{1\text{mol e}^-}{96485.3\text{C}} \right) \left(\frac{1\text{mol H}_2}{2\text{mol e}^-} \right) \left(\frac{6.02 \times 10^{23} \ \text{molecules H}_2}{1\text{mol H}_2} \right) = 3.12 \times 10^{15} \frac{\text{H}_2\text{/s}}{\text{cm}^2} \text{ per } \frac{\text{mA}}{\text{cm}^2} \tag{2}$$

The upper limit of the number of active sites was calculated based on the hypothesis that all cobalt atoms in the CoN_x/C catalyst formed active CoN_x centers and all of them were accessible to the electrolyte. The real number of active and accessible cobalt sites should be considerably lower than the calculated value. The bulk cobalt content of CoN_x/C revealed by the ICP-AES measurement was 0.14 wt%. Accordingly, the upper limit of active site density is

$$\frac{0.14}{100} \frac{\text{mg}}{\text{mg}} \times 2.0 \frac{\text{mg}}{\text{cm}^2} \times \frac{1}{58.93} \frac{\text{mmol}}{\text{mg}} \times 6.022 \times 10^{20} \frac{\text{sites}}{\text{mmol}} = 2.85 \times 10^{16} \ \text{sites cm}^{-2} \tag{3}$$

Particularly, at an overpotential of 100 mV, the HER current density is 3.57 mA per cm^2, and the TOF value of CoN_x/C was calculated to be

$$TOF = \frac{3.12 \times 10^{15} \times \frac{\text{H}_2\text{/s}}{\text{cm}^2} \text{ per } \frac{\text{mA}}{\text{cm}^2} \times 3.57 \frac{\text{mA}}{\text{cm}^2}}{2.85 \times 10^{16} \text{sites per cm}^2} = 0.39 \ \text{s}^{-1} \tag{4}$$

Similarly, the TOF value of CoN_x/C at an overpotential of 200 mV was calculated to be

$$TOF = \frac{3.12 \times 10^{15} \times \frac{\text{H}_2\text{/s}}{\text{cm}^2} \text{ per } \frac{\text{mA}}{\text{cm}^2} \times 59.3 \frac{\text{mA}}{\text{cm}^2}}{2.85 \times 10^{16} \text{sites per cm}^2} = 6.5 \ \text{s}^{-1} \tag{5}$$

Supplementary References

1. Le Goff, A. *et al.* From Hydrogenases to Noble Metal–Free Catalytic Nanomaterials for H_2 Production and Uptake. *Science* **326**, 1384-1387 (2009).
2. Andreiadis, E. S. *et al.* Molecular engineering of a cobalt-based electrocatalytic nanomaterial for H_2 evolution under fully aqueous conditions. *Nat. Chem.* **5**, 48-53 (2013).
3. Cobo, S. *et al.* A Janus cobalt-based catalytic material for electro-splitting of water. *Nat. Mater.* **11**, 802-807 (2012).
4. Jaramillo, T. F. *et al.* Identification of Active Edge Sites for Electrochemical H_2 Evolution from MoS_2 Nanocatalysts. *Science* **317**, 100-102 (2007).
5. Kibsgaard, J., Jaramillo, T. F. & Besenbacher, F. Building an appropriate active-site motif into a hydrogen-evolution catalyst with thiomolybdate $[Mo_3S_{13}]^{2-}$ clusters. *Nat. Chem.* **6**, 248-253 (2014).
6. Merki, D., Fierro, S., Vrubel, H. & Hu, X. L. Amorphous molybdenum sulfide films as catalysts for electrochemical hydrogen production in water. *Chem. Sci.* **2**, 1262-1267 (2011).
7. Kibsgaard, J., Chen, Z., Reinecke, B. N. & Jaramillo, T. F. Engineering the surface structure of MoS_2 to preferentially expose active edge sites for electrocatalysis. *Nat. Mater.* **11**, 963-969 (2012).
8. Lukowski, M. A. *et al.* Enhanced Hydrogen Evolution Catalysis from Chemically Exfoliated Metallic MoS2 Nanosheets. *J. Am. Chem. Soc.* **135**, 10274-10277 (2013).
9. Popczun, E. J. *et al.* Nanostructured Nickel Phosphide as an Electrocatalyst for the Hydrogen Evolution Reaction. *J. Am. Chem. Soc.* **135**, 9267-9270 (2013).
10. Popczun, E. J., Read, C. G., Roske, C. W., Lewis, N. S. & Schaak, R. E. Highly Active Electrocatalysis of the Hydrogen Evolution Reaction by Cobalt Phosphide Nanoparticles. *Angew. Chem. Int. Ed.* **53**, 5427-5430 (2014).
11. Chen, W.-F. *et al.* Hydrogen-Evolution Catalysts Based on Non-Noble Metal Nickel–Molybdenum Nitride Nanosheets. *Angew. Chem. Int. Ed.* **51**, 6131-6135 (2012).
12. Chen, W. F. *et al.* Highly active and durable nanostructured molybdenum carbide electrocatalysts for hydrogen production. *Energ. Environmen. Sci.* **6**, 943-951 (2013).
13. Zhao, Y., Kamiya, K., Hashimoto, K. & Nakanishi, S. Hydrogen Evolution by Tungsten Carbonitride Nanoelectrocatalysts Synthesized by the Formation of a Tungsten Acid/Polymer Hybrid In Situ. *Angew. Chem. Int. Ed.* **52**, 13638-13641 (2013).
14. Zheng, Y. *et al.* Hydrogen evolution by a metal-free electrocatalyst. *Nat. Commun.* **5**, 3783 (2014).
15. Duan, J., Chen, S., Jaroniec, M. & Qiao, S. Z. Porous C_3N_4 Nanolayers@N-Graphene Films as Catalyst Electrodes for Highly Efficient Hydrogen Evolution. *ACS Nano* **9**, 931-940 (2015).
16. Zou, X. *et al.* Cobalt-Embedded Nitrogen-Rich Carbon Nanotubes Efficiently Catalyze Hydrogen Evolution Reaction at All pH Values. *Angew. Chem. Int. Ed.* **53**, 4372-4376 (2014).
17. Tavakkoli, M. *et al.* Single-Shell Carbon-Encapsulated Iron Nanoparticles: Synthesis and High Electrocatalytic Activity for Hydrogen Evolution Reaction. *Angew. Chem. Int. Ed.* **54**, 4535–4538 (2015).
18. Vrubel, H. & Hu, X. Molybdenum Boride and Carbide Catalyze Hydrogen Evolution in both Acidic and Basic Solutions. *Angew. Chem. Int. Ed.* **51**, 12703-12706 (2012).
19. McKone, J. R., Sadtler, B. F., Werlang, C. A., Lewis, N. S. & Gray, H. B. Ni–Mo Nanopowders for Efficient Electrochemical Hydrogen Evolution. *ACS Catalysis* **3**, 166-169 (2013).
20. Gong, M. *et al.* Nanoscale nickel oxide/nickel heterostructures for active hydrogen evolution electrocatalysis. *Nat. Commun.* **5**, 4695 (2014).

21. Tran, P. D. *et al.* Copper molybdenum sulfide: a new efficient electrocatalyst for hydrogen production from water. *Energ. Environmen. Sci.* **5**, 8912-8916 (2012).
22. Wang, J. *et al.* Cobalt nanoparticles encapsulated in nitrogen-doped carbon as a bifunctional catalyst for water electrolysis. *J. Mater. Chem. A* **2**, 20067-20074 (2014).
23. Chen, Z. *et al.* Core–shell MoO_3–MoS_2 Nanowires for Hydrogen Evolution: A Functional Design for Electrocatalytic Materials. *Nano Lett.* **11**, 4168-4175 (2011).
